# An Improved, Bias-Reduced Probabilistic Functional Gene Network of Baker's Yeast, *Saccharomyces cerevisiae*

**DOI:** 10.1371/journal.pone.0000988

**Published:** 2007-10-03

**Authors:** Insuk Lee, Zhihua Li, Edward M. Marcotte

**Affiliations:** 1 Center for Systems and Synthetic Biology, Institute for Cellular and Molecular Biology, University of Texas at Austin, Austin, Texas, United States of America; 2 Department of Chemistry and Biochemistry, Institute for Cellular and Molecular Biology, University of Texas at Austin, Austin, Texas, United States of America; Columbia University, United States of America

## Abstract

**Background:**

Probabilistic functional gene networks are powerful theoretical frameworks for integrating heterogeneous functional genomics and proteomics data into objective models of cellular systems. Such networks provide syntheses of millions of discrete experimental observations, spanning DNA microarray experiments, physical protein interactions, genetic interactions, and comparative genomics; the resulting networks can then be easily applied to generate testable hypotheses regarding specific gene functions and associations.

**Methodology/Principal Findings:**

We report a significantly improved version (v. 2) of a probabilistic functional gene network [1] of the baker's yeast, ***Saccharomyces cerevisiae***. We describe our optimization methods and illustrate their effects in three major areas: the reduction of functional bias in network training reference sets, the application of a probabilistic model for calculating confidences in pair-wise protein physical or genetic interactions, and the introduction of simple thresholds that eliminate many false positive mRNA co-expression relationships. Using the network, we predict and experimentally verify the function of the yeast RNA binding protein Puf6 in 60S ribosomal subunit biogenesis.

**Conclusions/Significance:**

YeastNet v. 2, constructed using these optimizations together with additional data, shows significant reduction in bias and improvements in precision and recall, in total covering 102,803 linkages among 5,483 yeast proteins (95% of the validated proteome). YeastNet is available from http://www.yeastnet.org.

## Introduction

Gene networks provide a simple basis for organizing thousands of cellular components and their associations with each other, as well as for generating testable hypotheses about the components and the system as a whole. A number of research efforts have demonstrated that heterogeneous functional genomics and proteomics data can be integrated into gene (or protein) networks (e.g., [1]–[12]), thus organizing and relating highly complex data sets, as well as simplifying the prediction of new gene functions and associations on basis of the network connections. In such network integration approaches, relationships between genes are detected by various experimental or computational methods, and then combined in a bottom-up fashion in order to build a network model. As high-throughput biological experiments advance, we expect corresponding gains in network models derived from these data. Such improvements, however, are often tempered by the already extreme and growing complexity of the biological data.

There are three major problems in integrating diverse genomics data into network models. First, the genomics data are heterogeneous in their sensitivity and specificity for relationships between genes. For example, experimental methods such as mass spectrometry preferentially observe abundant proteins, while comparative genomics methods apply only to evolutionarily conserved genes. Increasing the sensitivity of detection usually carries a cost of increasing false positive identifications. Thus, the systematic bias for each method should be understood and considered during data integration. Second, genomics data sets vary widely in their utility for reconstructing gene networks. Thus, we need robust benchmarking methods that can evaluate each data set and allow comparison of their relative merits. Third, data sets are often correlated, complicating integration. However, the correlation can be difficult to measure because of both data incompleteness (a common problem) and sampling biases.

Probabilistic functional gene networks represent a class of gene network models that attempt to solve these problems, allowing integrative network models to be built from heterogeneous genomics data (e.g., [1], [3], [8], [10]–[13]). One key idea of such network models is the reinterpretation of genomics data as providing evidence for “functional coupling” between genes [1]. This non-mechanistic, but nonetheless useful, high level notion of gene association enables the integration of many different types of data, capturing diverse types of associations (e.g., direct physical interactions, regulatory interactions, membership in the same physical protein complex, etc.) precisely because the definition of gene association is inclusive. Such associations can be discovered using Bayesian statistical methods which allow robust evaluations to be made of functional associations between genes in a supervised learning framework, such as by measuring known pathways and cellular systems for their recapitulation by the data sets being analyzed. We previously reported such a probabilistic genome-wide gene network for yeast genes (dubbed YeastNet v.1) [1].

Here, we present optimized methods that improve our probabilistic functional gene network models. **Table 1** summarizes the major improvements. In particular, optimization of three major areas is highlighted, illustrating their effects on network quality. First, we reduced functional bias toward the dominant gold standard reference annotation during training. For example, most yeast gene functional annotation sets show biases towards genes of “protein biosynthesis” or “ribosomal proteins” [14], [15]. This bias inflates scores in a manner that does not generalize for other functions. Second, we apply a simple probability model for calculating confidence in protein physical interaction and genetic interaction data sets. We find the hypergeometric probability of an interaction occurring at random chance provides an excellent error confidence model for the interactions and simplifies their integration. Third, we introduce two thresholds that significantly improve the derivation of functional linkages from DNA microarray experiments. The combination of these improvements with additional data results in a markedly improved overall yeast gene network, spanning 95% of the validated yeast protein-coding genes. We demonstrate the network topology is predictive of essential genes, and apply the network to predict, then experimentally confirm, the function of the yeast gene *PUF6* in 60S ribosomal subunit biogenesis.

**Table 1:** A summary of major improvements to YeastNet version 2.

YeastNet v1 (Science 2004)	YeastNet v2 (This study)
34,000 linkages among 4,681 genes	102,803 linkages among 5,483 genes
Trained by KEGG pathway annotation	Trained by Gene Ontology biological process annotation
Training set includes linkages among biased term “Ribosome (KEGG:03010)”	Training set excludes linkages among biased term “Protein biosynthesis (GO:0006412)”
	Two new genome-wide complex mapping studies (Gavin *et al*. 2006, Krogan *et al*. 2006) were incorporated
	Probabilistic error model used to score functional linkages inferred from protein-protein interaction data
	Functional linkages inferred from “Gene neighbors” method were added
Genome-context approaches (Phylogenetic profiling, Rosetta Stone proteins) with 57 genomes	Genome-context approaches (Phylogenetic profiling, Rosetta Stone proteins, Gene neighbors) with 149 genomes
	Optimized methods inferring co-expression linkages including exclusion of gene pairs with potential cross-hybridization of cDNA and using two threshold parameters
Integration by weighted-sum with exponentially decaying weights for secondary evidence, optimized by one free parameter (D) determining decay rate	Integration by weighted-sum with linearly decaying weights for secondary evidence, optimized by two free parameters—D determining decay rate and T determining threshold of likelihood scores
Over-fitting was tested by independent annotation sets.	Over-fitting was tested by 0.632 bootstrapping
Additional functional linkages were inferred from network context (ContextNet)	Network context linkages did not improve network model, and were therefore omitted

## Results and Discussion

We incorporated three major improvements to the yeast probabilistic gene network, beyond inclusion of additional data sets: the reduction of bias in the reference training set, the introduction of probabilistic scores for physical and genetic interactions, and the introduction of filters to remove false-positive linkages from analysis of mRNA co-expression. We first discuss each of these improvements in turn, before demonstrating the overall quality of the network.

### Effect of a functionally biased reference set in learning a gene network from functional genomics data

The derivation of a probabilistic functional network from functional genomics and proteomics data using the log-likelihood strategy is an example of a supervised learning approach, distinguishing positive functional associations from negative associations on the basis of the performance of training associations in the data sets under analysis. The learning efficiency, however, is contingent upon the quality of the reference training sets, although the algorithms we employ are chosen for their robustness to false examples in the references. Learning efficiency also correlates with the extent of reference examples, as we cannot learn effectively using only a few examples. A third important characteristic of reference sets affecting supervised learning is the systematic bias among examples. In agreement with previous observations of yeast gene annotation [15], [16], we found that this last issue in particular was important for reconstructing a functional yeast gene network.

**Figure 1:**
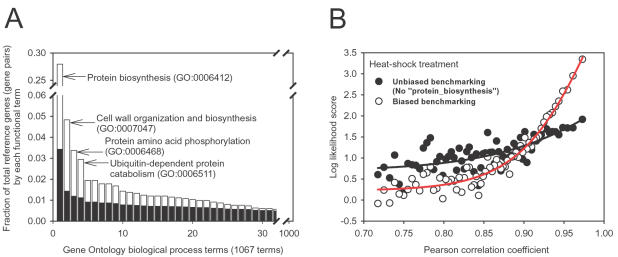
The effect of functionally biased Gene Ontology annotation on network training. (A) Frequency histograms of the usage of 1,067 Gene Ontology “biological process” annotations, ranked by the number of genes annotated with each term (black bars) and by the number of reference linkages derived using that term (white bars). Functional annotation is highly biased towards genes with the term “protein biosynthesis”. This functional bias becomes more severe in the reference linkages, given the combinatorial increase after linking all genes sharing a given term. As a result, linkages among protein biosynthesis genes compose >27% of total reference linkages. By contrast, the second most frequent term accounts for <5% of total reference linkages. (B) The likelihood of functional association between genes on the basis of the co-expression of their mRNAs across DNA microarray experiments (here, following heat-shock [19]) is significantly affected by the dominant reference term “protein biosynthesis”. For example, for the 1,000 most strongly co-expressed gene pairs, the likelihood of functional association between co-expressed genes is ∼30 fold higher than random chance (LLS∼3.4) (empty circles), but drops to ∼6 fold (LLS∼1.8) after masking the term “protein biosynthesis” in the reference set (filled circles). Thus, the high likelihood score from the biased reference set cannot be generalized to other functions. The black and red lines indicate sigmoid curve fits to the unbiased and biased reference analyses, respectively.

The most comprehensive and reliable functional annotation currently available for yeast is the Gene Ontology [17] annotation set. More than 70% of validated yeast protein-encoding genes are annotated by at least one of over 1,000 Gene Ontology “biological process” terms with support derived from reliable small-scale experimental evidence. Therefore, yeast Gene Ontology “biological process” annotation meets the first two requirements of a good reference set for efficient learning. However, the frequency distribution of annotation terms is heavily biased toward the single term “protein biosynthesis” (GO:0006412). This term alone is responsible for >27% of the total reference gene pairs (**Figure 1A**). We observed a similar bias in another widely used annotation set, The Kyoto Encyclopedia of Genes and Genomes (KEGG) [18] (data not shown).

There are many possible reasons for such biased annotation, ranging from bias in scientific interest—yeast has historically been a major model for studying many core cellular processes including eukaryotic protein biosynthesis—to bias in technological feasibility—it is generally easier to study highly expressed proteins such as ribosomal proteins—to intrinsic bias in the cellular system themselves—core molecular machines such as the ribosome legitimately incorporate more genes than many other cellular systems. We suspect that such bias is inevitable; nonetheless, we need to minimize its adverse effects for network reconstruction.

We examined the consequences of this bias by “masking” this dominant term in the annotation reference set, thereby removing all reference gene pairs linked *via* this term, and then testing data sets for their performance on the full and masked reference sets. For example, mRNA co-expression relationships between yeast genes across various heat-shock treatments [19] appear to strongly predict functional associations when benchmarked using the full, biased reference set (**Figure 1B**, open circles). However, that strong relationship largely disappears after masking only the single reference term “protein biosynthesis” (**Figure 1B**, closed circles). This observation clearly indicates that the strong functional associations derived from co-expression over these particular arrays are limited largely to protein biosynthesis genes. Thus, assigning a high likelihood score for gene pairs that co-express highly but are not in protein biosynthesis would be misleading. Examination of the frequency distribution of reference set gene pairs (**Figure 1A**) shows that the next most dominant term (“Cell wall organization and biosynthesis”, GO:0007047) accounts for <5% of reference pairs, with contributions from remaining terms decaying fairly smoothly. We therefore removed only the dominant “protein biosynthesis” term before reconstructing the probabilistic yeast gene network.

### Probabilistic inference of gene functional associations from physical protein-protein interactions and genetic interactions

Because of the generally strong correlation between protein physical or genetic interactions and functional associations, a map of such interactions among proteins is an invaluable source for learning about protein functions and pathways. Among many techniques of mapping protein physical interaction, yeast two hybrid assays and affinity purification followed by mass spectrometry have proved to be the most popular for their scalability. Two major genome-scale yeast two hybrid screens reported more than 4,000 binary interactions [20], [21]. While these interactions passed minimum quality criteria, we might not expect all to be equally informative for inferring functional associations. The original confidence measures—dividing interactions into a more reproducible “core” set and less reproducible “non-core” set [20]—is coarse-grained and may often miss functionally informative interactions.

Mass-spectrometry-derived interaction data, usually provided as a list of baits of affinity purification and their identified preys, is even more complicated for inferring binary physical or functional associations. Two different models of inferring binary interactions from the lists of identifications have been widely used—the spoke and matrix models [22]. The spoke model allows pair-wise relationships only between baits and preys in the same complexes, whereas the matrix model includes additional relationships inferred by pairing preys in the same complexes. These interpretative models exhibit different trade-offs between completeness and accuracy—the spoke model achieves high accuracy at the cost of incompleteness, whereas the matrix model provides a more complete model but relatively low accuracy due to pairing all prey proteins from a given bait with each other.

**Figure 2:**
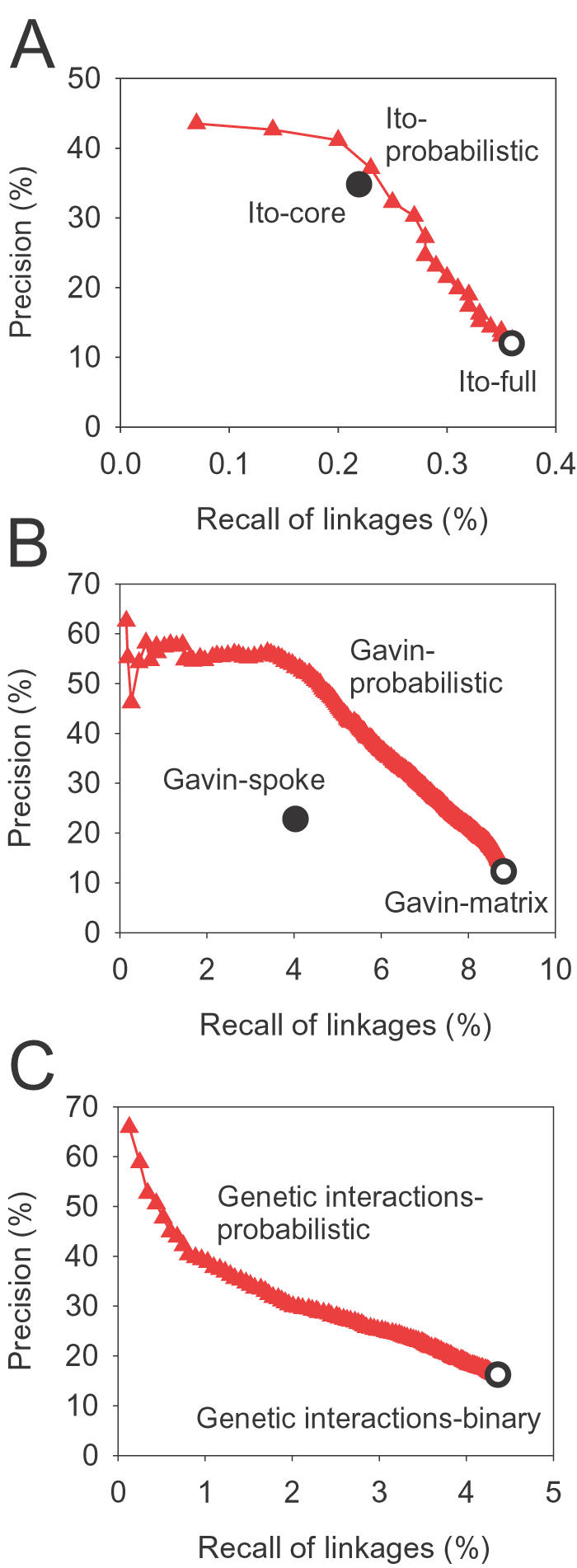
Assigning confidence scores to physical or genetic interactions. Performance of the hypergeometric probabilistic score is shown for gene functional associations inferred from (A) protein-protein physical interactions measured by the high-throughput yeast two hybrid (Y2H) screen of Ito *et al*. [20], (B) affinity-purified complexes identified by mass spectrometry by Gavin *et al*. [52], and (C) genetic interactions [43], [74]. Performance with the probability score is measured cumulatively for each successive bin of 200 interactions (A–C, red filled triangles), ranked by probability score. Recall and precision are calculated using the reference linkages derived from Gene Ontology “biological process” annotation masking the term “protein biosynthesis”. The Y2H core model described in [20] (A, filled circle) is more precise than the complete data set (A, open circle), but with reduced recall. Similarly, two different ways of inferring binary linkages from mass spectrometry-derived protein complexes [22]—the spoke (B, filled circle) and matrix models (B, open circle)—show differing trade-offs between precision and recall. The set of binary genetic interactions (C, open circle) shows very low precision for functional inferences, although the false positive rate of genetic interactions is generally perceived to be low; in contrast, the hypergeometric probability identifies a functionally informative subset of linkages. In general, the hypergeometric probability scores provide an excellent ranking of interactions in each of the data sets consistent with the linkages' functional informativeness.

A probabilistic model of protein-protein interactions should bypass the limitations of these coarse descriptive models, while providing higher resolution scoring important for data integration. We found that calculating the hypergeometric probability of the protein interactions occurring at random chance in a given data set generates a very well-behaved ranking of interaction accuracy in recall-precision analyses (**Figure 2A&B**). Note that this approach does not require training—instead, confidence is based only upon observations in the experiment under analysis and reflects the specificity with which a particular protein pair interacts, down-weighting promiscuous interactors and rewarding well-observed specific interactions. This scoring scheme outperforms the spoke model and attaches confidence values to each interaction in the matrix model, thereby separating high and low confidence matrix model interactions (**Figure 2A&B**). The hypergeometric score appears to work equally effectively for yeast two-hybrid and mass spectrometry interactions.

Interestingly, we also observed the hypergeometric probability-based confidence scores to effectively rank genetic interactions according to their utility for functional inferences (**Figure 2C**). Considering the likely low false positive rate of genetic interactions, this ranking probably does not reflect differences in the quality of interactions. Instead, it likely reflects the specificity of the genetic interactions, as each gene can participate in a varying number of genetic interactions and span a wide range of biological pathways [23]. For example, the yeast Hsp90 chaperone *HSP82* is considered to be a genetic capacitor genetically interacting with many (289) genes from diverse cellular processes, and may generically buffer phenotypic variation [24]. Since *HSP82* is a global modulator, the genetic interactions in which it participates only allow for weak function inferences. Therefore, the appropriate interpretation for the low precision observed for interactions involving *HSP82* is probably not that of false positives, but rather promiscuous interactions. Genetic interactions have been previously classified into within-pathway and between-pathway interactions [23], [25]; the hypergeometric probability model appears to rank within-pathway interactions above between-pathway interactions, thereby increasing the utility of genetic interactions for inferring functional associations between genes.

### Optimized method of inferring functional links by co-expression analysis

For inferring functional linkages from DNA microarray evidence, we employ the divide-test-integrate approach [1] for discovering functionally informative cases of mRNA co-expression. This method is in contrast to simply concatenating the results of all DNA microarray experiments to create a single, monolithic expression vector for each gene, then measuring correlation between these vectors. A co-expression network derived in this manner indeed shows a robust correlation between the extent of expression correlation and the degree of functional association, in part because of its high dimensionality. However, it generally works as a useful model only for limited groups of genes, such as consistently co-expressed housekeeping genes. The problem facing this method is that context-specific co-expression patterns evident in only a subset of experiments are overwhelmed by stochastic or uncorrelated expression changes across the remaining experiments. For example, consider the case of combining several experiments designed to detect expression dynamics during heat shock with a large number of unrelated experiments. Linkage between genes that respond coordinately under heat shock but not in the remaining experiments are unlikely to be detected in an analysis of the monolithic expression vectors. In contrast, linkages among these genes might be detected by the divide-test-integrate approach, in which each group of biologically coherent experiments is analyzed separately for co-expression linkages, followed by integration of linkages across the sets of experiments. However, its actual practice often entails increased false positive co-expression linkages because of lower dimension expression vectors and the correspondingly increased probability of observing such correlations at random.

For robust as well as sensitive co-expression linkage detection, we introduced two new parameters to filter false positive co-expression linkages. These filters operate by removing genes from the co-expression analysis that fail to show a minimum ratio of expression change (R) in a minimum number of microarray experiments (M), thereby eliminating the genes most likely to be unresponsive in the array set being analyzed. We optimized the choice of these two parameters for each set of array experiments by maximizing the area under a recall-precision curve (**Table 2**).

**Table 2:** SMD data sets used for co-expression links (total 500 experiments)

SMD category	N	R	M
Cell cycle	83	1.4	8
Diamide treatment	8	1.6	6
Diauxic shift	19	1.4	13
DNA damage response in mec mutant	19	1.8	4
DNA damage response in WT	19	1.4	7
DTT treatment	15	1.3	10
Heat shock treatment	31	1.8	16
Nitrogen limitation	9	1.6	7
Nutrition limitation (Leu, Ura, Phosphate, Sulfate)	100	1.0	4
Osmotic shock (hyper, hypo)	26	1.2	13
Oxidative stress with HP (H_2_O_2_)	40	1.5	14
Measuring the number of mRNA-associated ribosome	42	1.3	29
RNA decay measurement	58	1.9	36
YPD stationary culture	31	1.1	20

N: the number of experiments in the set

R: minimum absolute value of log base 2 ratio of expression between treated experiment and control

M: minimum number of microarray experiments that exceed the R threshold

**Figure 3:**
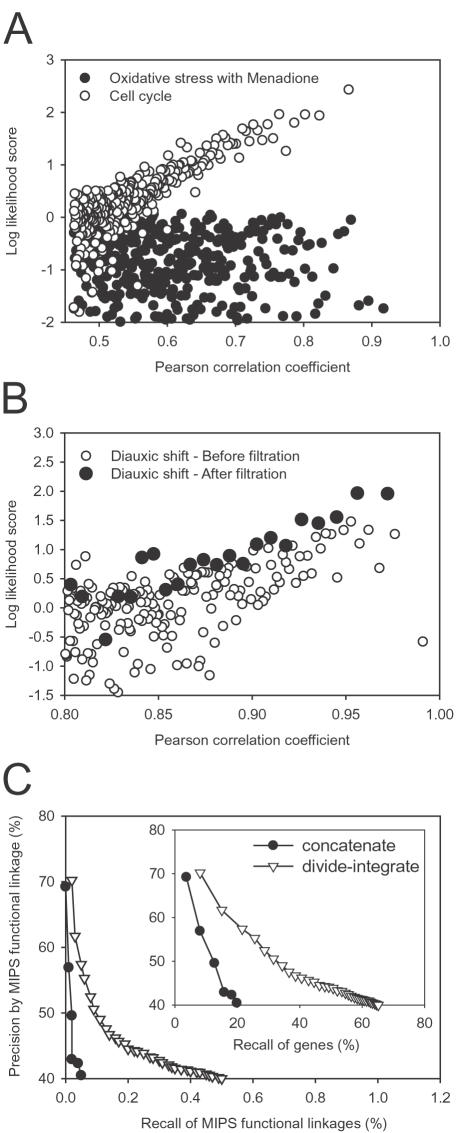
Optimizing the inference of linkages from mRNA co-expression. (A) Examples of a functionally informative DNA microarray data set and a non-informative one. Each set is illustrated as a scatter plot showing the log likelihood of functional association for each successive bin of 1,000 gene pairs (circles) ranked by decreasing Pearson correlation coefficient between expression vectors derived from that array set. The set of microarray data measuring oxidative stress responses following Menadione treatment [75] (filled circles) does not show a significant relationship between co-expression and the likelihood of functional association. In contrast, the set of cell cycle time course experiments [76] (open circles) shows a strong relationship. The effect of filtering genes using the parameters M and R is illustrated in (B). A data set of genes changing expression during the diauxic shift [77] (open circles) shows a noisy relationship between co-expression and the likelihood of functional association, especially for gene pairs with the highest Pearson correlation coefficients. However, by introducing the two threshold parameters, the relationship improves (filled circles), in particular decreasing variance considerably and improving the corresponding regression model. (C) The divide-test-integrate strategy [1] for inferring linkages, shown here calculated across all 500 microarray experiments (empty triangles) considerably outperforms analysis of the expression vectors constructed by concatenating the 500 experiments (filled circles). Precision is measured using reference linkages derived from MIPS functional annotation, masking the term “protein synthesis”, and recall is calculated for either reference linkages or total yeast genes (inset).

**Figure 4:**
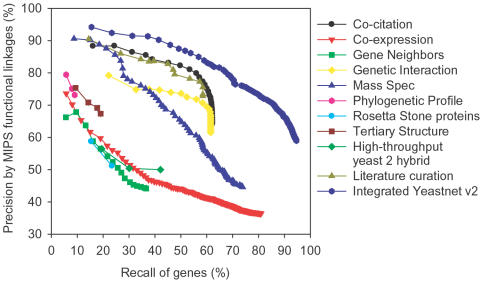
Summary of benchmarking for the YeastNet v. 2 integrated functional gene network, along with the functional linkage sets derived from the ten individual types of data. Precision and recall of yeast genes are calculated using the unbiased MIPS functional linkage reference set, as in Figure 3C. Gene pairs in each set were ranked by LLS scores, then cumulative precision and recall were calculated for each successive bin of 1,000 gene pairs (each symbol indicates a bin). The confidence measures for each individual data type can be seen to rank the gene pairs effectively. YeastNet v. 2 shows high overall performance, with the integration of the heterogeneous genomics data improving both reliability and completeness of the overall model.

Beyond filtering genes, we also removed entire data sets that proved uninformative for reconstructing a functional network: We measured the relationship between the degree of co-expression between two genes, measured as the Pearson correlation coefficient (PCC) of their expression levels across the arrays under consideration, and the likelihood of their functional association, measured by the log likelihood of belonging to the same pathway (LLS, see Methods) between the genes in each successive bin of 1000 gene pairs ranked in descending order by PCC. Across 18 total sets of DNA microarrays from SMD [26], containing 581 individual array experiments, we found 14 sets showed a significant relationship (e.g., Cell cycle; **Figure 3A**) and 4 sets showed no relationship (e.g., Oxidative stress with Menadione; **Figure 3A**), as listed in **Tables 2** **and** **3**. Alternate measures of expression correlation (the non-parametric Spearman rank coefficient and mutual information measures) failed to improve performance over PCC. Filtering the unresponsive genes as described above further improved the relationships, as shown for an example in **Figure 3B**. In order to ensure representation of housekeeping genes, the 14 informative array sets were also concatenated into monolithic expression vectors spanning 500 experiments and analyzed for co-expression linkages as above. The benefits of the divide-test-integrate method are illustrated in the improved precision for any given coverage of genes or reference linkages, as shown in **Figure 3C** on the independent MIPS protein functional linkage reference set (excluding the term “protein synthesis”).

**Table 3:** SMD data sets tested but rejected (total 81 experiments)

SMD category	N
Calcium treatment	24
Oxidative stress with Menadione	30
Salt treatment	18
Zinc treatment	9

N: the number of experiments in the set

### Assessment of YeastNet version 2 as a predictive model

In total, ten types of functional genomics, proteomics, and comparative genomics data sets are integrated into the network (**Table 4**), as described in the Methods section (see the pseudo-code for an overview of the procedure). Approximately 1,800,000 individual experimental observations were integrated into the network model, optimizing a total of ∼155 free parameters in order to construct the network. Using a permissive scoring threshold corresponding to the log likelihood score (LLS>0.916) of non-core genome-wide Y2H screens [20], YeastNet v. 2 contains a total of 102,803 linkages covering 5,483 yeast proteins (covering >95 % of validated yeast proteome).

**Table 4:** Ten genomics data types incorporated into YeastNet version 2

Linkage set	Raw data sources	N	E
Co-citation	29,135 PubMed abstracts for *S*. *cerevisiae*	3,605	29,483
Co-expression	500 *S*. *cerevisiae* microarray experiments from Stanford Microarray Database [26]	2,923	31,543
Gene Neighbor	BLAST hits for 133 completely sequenced archaeal and bacterial genomes	1,098	4,961
Genetic Interaction	MIPS genetic interactions and large-scale synthetic lethal screening [74]	3,556	12,538
Affinity purified complex mapping by mass spectrometry	Three large-scale mass spectrometry analyses of affinity purified complexes [52]–[54]	3,368	31,931
Phylogenetic Profile	BLAST hits for 117 completely sequenced bacterial genomes	351	1,050
Rosetta Stone proteins	BLAST hits for 149 completely sequenced genomes	801	856
Literature curation	Protein interactions supported by small scale experiments collected by manual literature curation, and deposited into BioGRID [29] and DIP [30]	3,390	11,728
Inferred interaction from protein tertiary Structure	Prediction of physical interaction based on protein tertiary structure data [72]	1,092	3,405
High-throughput yeast 2 hybrid	Five large-scale yeast 2 hybrid screens [20], [21], [55]–[57]	1,792	2,055

N : Total number of genes incorporated into the integrated YeastNet version 2

E : Total number of linkages incorporated into the integrated YeastNet version 2

The integrated model, along with the various sets of linkages derived from individual data sets, was assessed on an independent test set of gene functional linkages derived from the MIPS protein function annotation set, calculating recall and precision of the MIPS reference linkages (**Figure 4**). For this purpose, we measure recall of genes, rather than gene pairs, in order to assess the generality of predictions across the entire genome. The integrated network shows high gene coverage and high precision across the entire network. As expected, the integrated network surpasses any individual data set for precision at a given coverage; the complete network covers >95% of the protein-coding genes in the yeast genome with >60% precision on the inferred linkages (*i.e*., at least 6 out of 10 predicted linkages are true).

**Figure 5:**
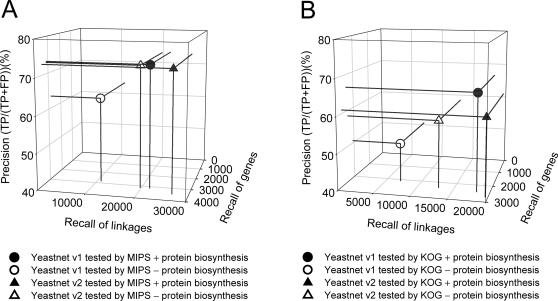
YeastNet v. 2 outperforms YeastNet v. 1 [1], as measured by precision and recall of either genes or reference linkages on independent reference sets. (A) shows performance on the MIPS functional linkage reference set, with or without masking the term “protein synthesis”, while (B) shows performance on reference linkages derived from KOG functional categories. For both reference sets, we observe significantly improved recall by YeastNet v. 2 for both yeast genes and reference linkages. The effect of the annotation “protein synthesis” is revealed by a significant drop of precision in YeastNet v. 1 but not v. 2 after masking the term during benchmarking. The higher fraction of linkages derived from “protein synthesis” in KOG (∼17% of total linkages) than MIPS (∼4% of total MIPS linkages) explains the apparently higher precision of v. 1 than v. 2 when including the term in (B), resulting in a correspondingly larger drop in precision of v. 1 when the term is masked. All analyses in (A) and (B) are for the 34,000 most confident linkages of each network.

We compared the overall performance of YeastNet version 2 to that of YeastNet version 1 by recall-precision analysis on the independent test sets. We previously defined a confident sub-network by taking only the top 34,000 functional linkages (covering 4,681 yeast proteins) [1] and used that for detailed biological interpretation. We therefore selected the top 34,000 linkages of both versions of YeastNet in order to perform a fair comparison. This subset of YeastNet v. 2 covers 4,649 yeast proteins (>80% of the validated yeast proteome). In tests of the MIPS functional linkage reference set that included linkages derived using the functional category “protein synthesis”, the precision of the two networks is comparable, while coverage—for both genes and reference linkages—is significantly improved for the new network model (**Figure 5A**). Superiority of the new network becomes more obvious when we mask the reference linkages derived from the term “protein synthesis”. Precision of the new network is minimally influenced by masking of this single term. In contrast, YeastNet v. 1 shows a noticeable drop in precision, indicating a bias towards protein synthesis-related functions. We observe the same trend using another independent functional linkage reference set derived from KOG functional categories (**Figure 5B**), with the precision of YeastNet v. 2 changing only minimally with removal of the KOG reference term “protein synthesis”, while precision of YeastNet v. 1 drops below v. 2. Roughly 17% of total KOG reference linkages are derived from the annotation term “protein synthesis”, while only 4.3% of total MIPS linkages are, accounting for the larger effect seen on the KOG benchmark. (The effect is also accentuated by the fact that MIPS annotates 3,752 yeast genes, whereas KOG annotates only 3,022.) Therefore, we conclude that the new network, YeastNet v. 2, is a significantly improved gene functional network, both showing higher accuracy and coverage, as well as better generalization to a more diverse set of cellular systems.

**Figure 6:**
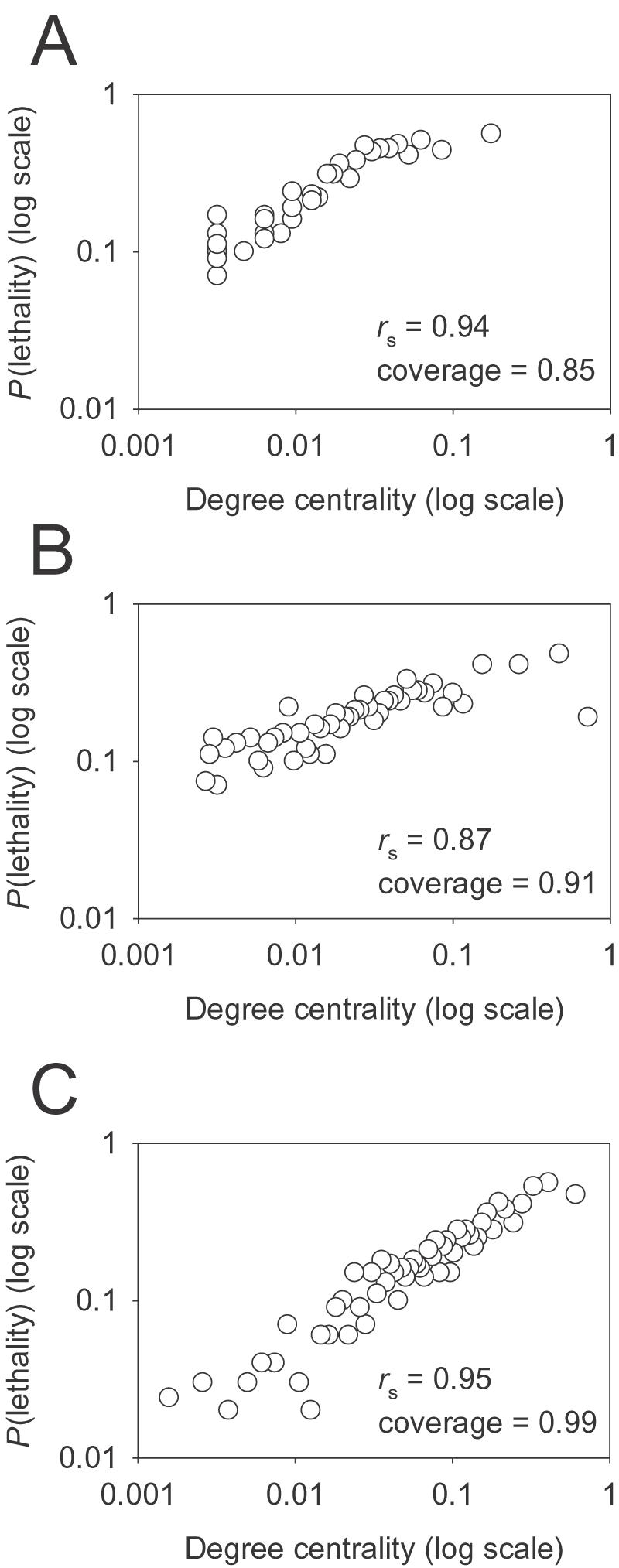
YeastNet v. 2 shows improved correlation between gene centrality and lethality. Each plot presents the correlation (for a given network) between network centrality, calculated as the number of interactions per gene normalized by the maximum observed value, versus the essentiality of the genes, calculated as the fraction of essential yeast genes [27] for each successive bin (open circle) of 100 genes ranked by decreasing degree centrality. (A) shows the trend for a high quality protein-protein physical interaction network derived from DIP [30] and bioGRID [29], (B) shows the trend for YeastNet version 1 (34,000 most confident linkages only), and (C) shows the trend for YeastNet version 2. For both functional networks, degree centrality is weighted by the interaction LLS scores (*i.e*., calculated as the sum of LLS scores for a gene, divided by the maximum sum of LLS scores observed in the network). The degree of correlation is measured as the Spearman rank correlation coefficient (*r*_s_).

Another aspect of the predictive quality of a gene network relates to an observed correlation between a gene's tendency to be essential [27] and its centrality in a network, measured as the number of interactions in which the gene participates. This correlation was initially observed for the yeast physical protein-protein interaction network [28]. Consistent with the original observation, the high quality physical protein-protein interactions derived from small-scale experiments (here, collected from bioGRID [29] and DIP [30]) show a strong correlation between degree centrality and lethality (Spearman rank correlation (*r*_s_)  =  0.94; **Figure 6A**). YeastNet v. 1 also showed a strong correlation—slightly worse in quality than the physical interactions but covering a higher proportion of the experimentally identified essential genes (increasing from 85% coverage in the protein-protein interaction network to 91% by YeastNet v. 1; **Figure 6B**). One explanation for this slightly lower correlation is that linkages in YeastNet v. 1 are enriched among genes of protein biosynthesis, especially ribosomal proteins, because of the biased reference set. This trend would lower the correlation, as only ∼18% of yeast ribosomal genes (defined by GO cellular component annotation) are essential, similar to the general background proportions of essential genes [27]. Consistent with this notion, we found that YeastNet v. 2 shows a higher correlation between degree centrality and lethality (Spearman rank correlation (*r*_s_)  =  0.95) while covering nearly all (99%) of the experimentally identified essential yeast genes (**Figure 6C**). This indicates that the optimized learning methods, considering functional bias, produce a more globally predictive network model.

### Experimental validation of the top ribosome biogenesis prediction, *PUF6*

In addition to the above computational validation, we also experimentally validated predictions arising from the new gene network. Using the new network, we predicted new genes to be involved in the process of ribosomal biogenesis, which is a fundamental process critical for cells and widely conserved across eukaryotes. New ribosomal biogenesis genes were inferred by identifying close network neighbors to the known ribosomal biogenesis genes. Specifically, we generated a seed set of known ribosome biogenesis genes based on their Gene Ontology biological process annotation (*n*  =  238 yeast genes annotated by the terms “ribosome assembly”, “rRNA”, or “35S”), then prioritized their network neighbors by the sum of their LLS scores to genes of the seed set. This list of genes was filtered to remove known ribosomal proteins. **Table 5** lists the top 5 predictions. Two of the top 5 genes, *CIC1* and *ESF2* have been verified in the literature [31], [32] but had not yet been included in the ribosome biogenesis annotation set we employed, and thus can be considered true predictions already verified by published studies. Moreover, these predictions are also supported by multiple lines of evidence including inferred functional linkages based on high-throughput data (e.g., co-expression and mass spectrometry analysis; **Table 5**). All five genes are known to be localized to the nucleolus [33], strongly supporting a possible role in ribosome biogenesis.

**Table 5:** Top five predictions of new ribosomal biogenesis genes

Rank	Gene	Evidence	GO description
1	*PUF6* *	CX, MS, LC	nucleus, nucleolus, regulation of transcription, mating-type specific
2	*CIC1* *	CX, MS, LC	proteasome complex (sensu Eukaryota), nucleolus, protein catabolism
3	*KRE33*	MS, CX, LC	nucleolus
4	*ESF2* *	CX, MS, LC, GT	nucleolus, cytoplasm
5	*BFR2*	CX, MS, LC, YH	nucleolus, ER to Golgi transport

* Experimentally validated by this study and others.

CX: co-expression, GT: genetic interaction, LC: literature curation, MS: mass spectrometry complex analysis, YH: genome-scale yeast two hybrid

We selected the top-ranked prediction, *PUF6*, for experimental validation. *PUF6* encodes an RNA-binding protein previously known to be involved in mating-type determination *via* its translational repression of *ASH1* mRNA prior to *ASH1* mRNA localization to the bud tip [34]. While previous computational evidence associates *PUF6* with ribosomal biogenesis [35], there is not yet direct experimental support for its involvement. We therefore experimentally tested *PUF6* for its participation in ribosomal biogenesis.

**Figure 7:**
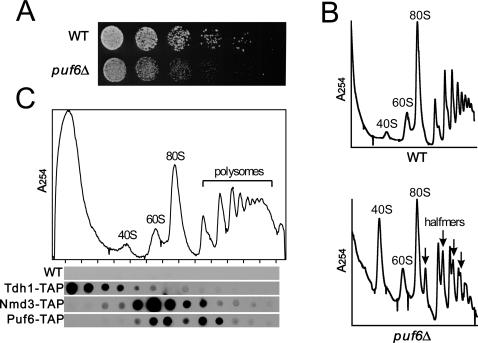
Experimental validation of the participation of *PUF6* in 60S ribosomal subunit biogenesis. (A) A *puf6Δ* deletion strain [27] shows a marked conditional growth defect compared to wild-type (WT) cells when grown at 20 °C, accompanied by (B) a decrease in the ratio of 60S/40S ribosomal subunits and the formation of halfmer polysomes, as measured by monitoring absorbance at 254 nm of clarified yeast cell lysate separated on a 7 to 47 % sucrose density gradient. Additional evidence for the participation of *PUF6* in 60S biogenesis can be seen (C) in the co-sedimentation of the TAP-tagged Puf6 protein [40] with the 60S ribosomal subunits, as measured by Western blotting of lysates separated by sucrose density gradient. *TDH1* encodes a cytoplasmic protein with no known association with ribosomal subunits, serving as a negative control. *NMD3* encodes a known 60S biogenesis factor [38], serving as positive control. Puf6-TAP shows a similar sedimentation profile as Nmd3-TAP, supporting the role of *PUF6* in 60S ribosomal subunit biogenesis.

We might expect yeast strains defective in ribosomal biogenesis to show a slow growth phenotype; we tested a *puf*6Δ deletion strain [36] and indeed observed significant growth retardation compared to the wild-type strain when cultured at 20 °C (**Figure 7A**). We then analyzed the polysome profile of the deletion strain in order to assess defects in ribosome processing consistent with a biogenesis defect. We observed an abnormal decrease in the ratio of 60S/40S ribosomal subunits and detected the presence of halfmers in the *puf*6Δ deletion strain (**Figure 7B**). Such halfmers—shoulders on the 80S and polysome peaks—arise from mRNA bound by an extra 40S subunit stalled at the AUG initiation codon and are characteristic of 60S subunit biogenesis defects and blockage of translation initiation at the 60S subunit joining stage (e.g., [37]–[39]). Both the decrease in 60S subunit abundance relative to 40S and the presence of halfmers indicate a probable role of *PUF6* in 60S ribosomal subunit biogenesis. We further tested the participation of *PUF6* in 60S biogenesis by performing Western blot analysis on an epitope-tagged version of the Puf6 protein [40]. We observed the epitope-tagged Puf6 protein to co-sediment in a sucrose gradient with the 60S ribosome in a fashion similar to the known 60S ribosome biogenesis factor Nmd3p [38] (**Figure 7C**). Therefore, the top network prediction for proteins most likely to participate in ribosome biogenesis could be experimentally confirmed. In all, 3 of the top 5 predictions could be directly confirmed, with the remaining 2 highly likely given their nucleolar localization.

### Conclusions

In this study, we present several optimizations that significantly improve the predictive power of a probabilistic functional gene network of yeast. There are three major aspects worth noting. First, our current functional genomics knowledge is severely biased. This bias leads to biased learning unless appropriately taken into account, as the effect of reference linkages from the dominant GO term “protein biosynthesis” is quite strong (**Figures 1** **and** **5**). Second, physical protein interaction and genetic interaction data can be assigned scores that allow, on a per interaction basis, for fine-grained, continuous valued confidence measures. The score that we employ, based on the hypergeometric probability, is simple and robust, and works across a variety of different experimental techniques, and would therefore even be appropriate as a final confidence score directly out of large-scale experimental assays (e.g., as in [41]). Introduction of this score significantly improves the performance of these data in deriving the probabilistic gene network. Third, introducing two additional parameters into the analysis of mRNA co-expression linkages significantly decreases the number of false positive linkages while simultaneously decreasing the variance in the quality of the derived linkages (**Figure 3B**). Incorporation of each of these optimizations into YeastNet v. 2 significantly improves the quality of the model, improving precision and recall on independent test sets and increasing generality of the model for more diverse cellular systems. We expect that the protocol we present for calculating the network is general and could be applied to other organisms essentially directly as described.

We describe applications of the gene network for functional prediction (prediction of ribosomal biogenesis genes) and prediction of essential genes. In order to perform similar analyses of YeastNet v. 2, we have established a web site (http://www.yeastnet.org) where the network can be downloaded in full. We anticipate posting future updates of the network to this site as new data sets become available.

## Materials and Methods

### *Saccharomyces cerevisiae* gene set

YeastNet version 2 is based on the verified 5,794 protein encoding open reading frames (ORFs) of the yeast genome downloaded from *Saccharomyces cerevisiae* Genome Database (SGD) [42] on March 2005. All linkages and calculations of genome coverage are based on this gene set.

### Reference and benchmark sets

In order to benchmark the assigned functional linkages in this study, three different reference sets were used. As a major reference set for benchmarking, we used the Gene Ontology (GO) annotation, downloaded from the *Saccharomyces cerevisiae* Genome Database (SGD) [17] on March 2005. The GO schema lists three hierarchies of function describing “biological process” (i.e., pathways and systems), “molecular function” (i.e., biochemical activities), and “cellular component” (i.e., subcellular localization). For training the network, we used the *Saccharomyces cerevisiae* GO “biological process” annotation, which contains up to 14 different levels of information under the term “biological process” within the hierarchy. We used terms belonging to levels 2 through 10. We also excluded the term “protein biosynthesis” because it annotates so many genes as to significantly bias the benchmarking. To construct the reference set of linkages, we considered all gene pairs as functionally linked if they shared annotation from this set of GO terms. These pairs comprised our positive reference set for training network models. Negative examples were constructed as pairs of annotated genes not sharing any annotation terms, *i.e*., all other links among this annotated set of genes.

Specifically, 66,174 positive reference pairs were employed, representing all gene pairs sharing any GO biological process terms between levels 2–10 (except for the biased term “protein biosynthesis”). These pairs are provided on the supporting web site (http://www.yeastnet.org). All other pairs of these genes were implicitly defined as the negative reference pairs. For example, the genes *NOP1* and *SIK1* represent a positive example, sharing the GO terms ‘rRNA modification’, ‘35S primary transcript processing’, ‘processing of 20S pre-rRNA’. The genes *BUD5* (‘bud site selection’, ‘pseudohyphal growth’, ‘small GTPase mediated signal transduction’) and *NOG1* (‘ribosome-nucleus export’) are annotated, but do not share terms, and represent a negative example.

We also employed two independent functional linkage reference sets for testing functional linkages. One was derived from the Munich Information Center for Protein Sequences (MIPS) [43] protein function annotation. We used the 11 major categories from the top level MIPS functional category annotation. The second reference set was derived from the clusters of orthologous group (COG) annotation [44], which is based on reconstructing homologous groups of proteins in such a manner as to considerably enrich for orthologous proteins within each group, with the functions of genes assigned within 23 broad categories (such as “Transcription” and “Signal Transduction Mechanisms”) based on the well-annotated proteins with each COG. We use the version of COG that includes multicellular eukaryotic genomes (named eukaryotic orthologous groups, or KOG) [45]. Positive and negative linkage sets were constructed from each of these reference sets as for the GO set.

### Benchmarking and integrating heterogeneous functional genomics data

Different types of genomics data sets differ considerably in their utility for inferring functional linkages. We standardized the contributions from heterogeneous genomic data sets by scoring using the log likelihood score (*LLS*) scheme previously described in [1].

In this scheme, the score for each data set (or subset; e.g., a set of gene pairs co-expressed to a certain extent) is calculated as
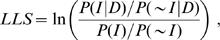
where *P(I|D)* and *P(∼I|D)* are the probabilities for gene pairs linked by the given data *(D)* to share *(I)* or not share *(∼I)* functional annotation, respectively, and *P(I)* and *P(∼I)* represent the prior probabilities of sharing/not sharing functional annotation, respectively. For estimating the conditional probabilities *P(I|D)* and *P(∼I|D)*, we calculated the fraction of annotated gene pairs in the data set being analyzed that were found in the positive or negative reference sets, respectively. *P(I)* and *P(∼I)* were calculated as the overall frequencies of positive reference pairs (annotated gene pairs sharing annotation) and negative reference pairs (annotated gene pairs not sharing annotations). Thus, an *LLS* score of zero indicates that the data is no more informative than random expectation for discovering functional linkages; increasingly positive LLS scores indicate increasing information in the data set for discovering functional linkages.

To avoid overtraining, we employed 0.632 bootstrapping [46], [47] for all *LLS* calculations. 0.632 bootstrapping has been shown to provide a robust estimate of classifier accuracy, out-performing cross-validation [48], especially for very small data sets (e.g., see [49]), and is thus appropriate even for more poorly annotated genomes. Unlike cross-validation, which uses sampling without replacement for constructing test and training data sets, 0.632 bootstrapping employs sampling with replacement, constructing the training set from data sampled with replacement and the test set from the remaining data that weren't sampled. Each linkage has a probability of 1-1/*n* of not being sampled, resulting in ∼63.2% of the data in the training set and ∼36.8% in the test set [50]. The overall *LLS* is the weighted average of results on the two sets, equal to 0.632*LLS_test_ + (1-0.632)*LLS_train_.

For data sets in which each gene pair is associated with a continuous score (e.g., correlation coefficient, mutual information, etc.), we calculated *LLS* scores for bins containing equal numbers of gene pairs. Those *LLS* scores and their corresponding data scores (the mean data scores for a bin) were used to calculate regression models (see **Figure 1B** for examples), which were then used to map individual data intrinsic scores to *LLS* scores in a continuous manner, allowing calculation of LLS scores for gene pairs lacking annotation. In general, quadratic curve fits tended to overscore gene pairs with the highest data-intrinsic scores (e.g., those with the highest correlation coefficients) during the extrapolation to unannotated genes; sigmoidal fits provided equivalent quality regression models, but were more conservative for the highest scoring cases.

For integrating *LLS* scores from different data sets, we employed the weighted sum method [1] in order to take into account correlations among the data sets. The published weighted sum method was modified by using linearly decaying weights for additional datasets, and by including a new free parameter, *T*, which represents a minimum *LLS* threshold on the data sets being integrated. The weighted sum (*WS*) integrating multiple likelihood scores of functional association for a gene-pair was calculated as:
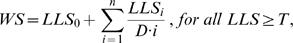
where *LLS_0_* represents the maximum *LLS* score for a given gene pair, *D* is a free parameter determining the decay rate of the weight for secondary evidence, and *i* is the rank order index of *LLS* scores, ranking gene pairs starting from the second highest *LLS* with descending magnitude for all *n* remaining *LLS* scores. For integration, we consider only *LLS* scores above the threshold *T*, thereby excluding noisy low scoring linkages. The free parameter *D* ranges from 1 to +∞, and is optimized to maximize overall performance (measured as the area under a recall-precision curve) of the integrated model. As the optimal value of *D* approaches +∞, *WS* approaches *LLS_0,_* and lower scoring *LLS* scores do not provide any additional likelihood, as appropriate when all data sets are completely dependent. We independently explicitly test the performance of a *naïve* Bayesian integration of the *LLS* scores (here, simply the sum of the *LLS* scores for a given gene pair), then select the integration approach maximizing the area under a plot of *LLS* versus gene pairs incorporated in the network.

Regarding the choice of linear versus exponential decay of confidence in secondary evidence, we observe better performance (measured by recall-precision analysis) using the linear model when accompanied by more extensive secondary evidence and improved filtering of false positive linkages prior to integration. In YeastNet v.1, more low-scoring false-positive linkages were incorporated, and their contributions as secondary evidence were more strongly down-weighted under the exponential model. However, in YeastNet v. 2, new filters (in particular, new probabilistic scores for protein interactions and the introduction of thresholds for DNA microarray data) down-weight or remove many false positive associations prior to integration. The addition of new data sets also has the effect of increasing the quantity of secondary evidence. Thus we empirically observe that as secondary lines of evidence become more available and informative, the linear dependency model performs better.

### Inferring gene functional linkages from mRNA expression data

Gene functional linkages were inferred from mRNA expression data deposited in the Stanford Microarray Database (SMD) by July 2005 [26]. Co-expression relationships were measured as the Pearson correlation coefficient (PCC) between pairs of genes' mRNA expression vectors, accepting only PCC values statistically significant at the 99% confidence level by t-test. From the set of gene pairs with significant PCC scores, we excluded pairs with cDNA sequence homology (defined as a BLAST E-value<10^−4^ and percentage nucleotide sequence identity >70% over the aligned regions [51]) in order to reduce false positive co-expression linkages caused by cross-hybridization on the DNA microarrays. As demonstrated previously [1], overall recall/precision of expression-derived linkages can be improved by analyzing subsets of arrays independently, rather than as a single composite expression vector. We tested a total of 581 DNA microarray experiments comprising 18 sets, as defined by SMD (**Tables 2** **and** **3**). We found that 14 SMD sets, containing a total of 500 array experiments, exhibited a significant correlation between PCC and the log likelihood score; we considered only these data sets further.

We introduced two additional parameters to improve co-expression inferences: a threshold for the minimum observed change in mRNA levels across the set of array experiments (R in **Table 2**), and a threshold for the minimum number of microarray experiments with expression values greater than R (M in **Table 2**). Thus, only genes that are differentially expressed by at least R-fold (in either direction) on at least M microarrays in the given data set will be considered for co-expression linkages. These parameters considerably reduce the linkage false positive rate by removing genes that do not vary across the set of arrays being analyzed, under the premise that genes that are expressed at a constant level across the tested conditions are not likely to be relevant to the conditions of the experiments or to participate in strong co-expression relationships. These filters therefore remove false positive linkages derived from experimental noise and drift in otherwise unchanging baseline expression levels. We optimized the two thresholds for each set of SMD arrays, maximizing the area under a curve plotting the number of genes incorporated in the inferred linkages versus cumulative log likelihood score of the linkages (**Table 2**).

In order to include otherwise robust co-expression linkages missed by these analyses, we also concatenated all 500 experiments derived from the 14 selected SMD data sets and derived co-expression linkages from these concatenated expression vectors. These linkages plus those from each of the 14 SMD subsets were integrated by the weighted sum method.

### Inferring gene functional linkages from experimental protein-protein interaction data

Physical protein-protein interactions (PPI) and genetic interactions (GI) were collected from the Database of Interacting Proteins (DIP) small-scale experiment set (downloaded March 2003) [30], BioGRID (downloaded on June 2006) in which all interactions are supported by literature curation [29] and literature collection by MIPS [43]. These interactions are highly confident, because genetic interaction screens inherently provide low false-positive rates (Type I errors), and all physical interactions in these sets are derived from small-scale studies. Additional physical interactions were collected from published genome-scale screens using mass spectrometry analyses of affinity-purified protein complexes [52]–[54] or high throughput yeast two hybrid (Y2H) assays [20], [21], [55]–[57].

We applied a quantitative error model developed for PPI data sets [41], [58] in order to assign probabilistic confidence scores to each PPI or GI gene pair. Instead of modeling simple binary bait-prey interactions for yeast two hybrid assays, inferred binary interactions from mass spectrometry analysis of affinity-purified protein complex [22], or binary genetic interactions, we calculated the hypergeometric probability of interaction between two proteins by random chance, assigning a probability (*p*-value) to the pair as:
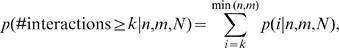
where
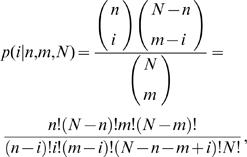
and where *k* is the number of interactions observed between proteins A and B in the complete interaction data set (*e.g*., depending on the data set, counting the number of yeast two-hybrid interactions, mass spectrometry co-purifications, or genetic interactions involving both A and B), *n* is the number of observed interactions involving protein A, *m* is the number of observed interactions involving protein B, and *N* is total number of experiments with ≥1 interaction measured (*e.g*., depending on the data set, counting the number of total detected yeast two-hybrid interactions, the number of pull-down experiments with at least one interaction prey identified by mass spectrometry analysis, or the number of total detected genetic interactions). Using this measure, interactions between proteins with many different interacting partners (*i.e.*, frequent interactors) have a high probability of occurring by random chance, indicating either promiscuous or strongly context-dependent interactions. Given probable association with many other partners, such cases therefore receive a correspondingly low confidence in the gene pair's specific interaction with each other.

### Inferring gene functional linkages from genome context

We employ three genome context methods for inferring functional linkages from genome sequences: phylogenetic profiling (PG) [59]–[61], the Rosetta Stone protein (RS) (or gene-fusion) method [59], [62]–[64], and gene neighbors [3], [65], [66]. Linkages for each method were derived from analysis of a database of 149 genomes (117 bacteria, 16 archaea, and 16 eukaryotes).

Briefly, each yeast protein sequence was compared to every other sequence using the program BLASTP with default settings [67]. Rosetta Stone linkages and gene neighbor linkages were calculated from these comparisons as in [68] and [3], respectively. Phylogenetic profiles were constructed from these comparisons and analyzed as in [69] with the following modifications. We found the profiles corresponding to major phylogenetic groups of organisms varied widely in their utility for deriving functional gene associations. In particular, inclusion of eukaryotic and archaeal genomes did not significantly improve performance. Instead, we found the best performance—measured as the performance maximizing the area under a plot of LLS versus the number of genes participating in the linkages—by inferring functional linkages from a profile constructed only from bacterial genomes. For discretizing BLAST E-values prior to calculation of mutual information between phylogenetic profiles, we binned by equal numbers of examples rather than by equal intervals of E-values, accounting for the non-uniform distribution of BLAST E-values. We observed the best results from using 3 bins.

### Inferring gene functional linkages from literature mining

We identified functional linkages by mining the scientific literature (specifically, Medline abstracts) using the co-citation approach [70], [71] as in [1]. We analyzed a set of *N*  =  29,135 Medline abstracts that included the word “*Saccharomyces cerevisiae*” in the abstract for perfect matches to either the standardized names or common names (or their synonyms) of 5,794 yeast genes.

### Inferring gene functional linkages from protein tertiary structure

Functional linkages were also inferred from physical interactions predicted between proteins pairs based upon modeling their 3-dimensional structures into *X*-ray crystal structures of homologous protein complexes. We used the tertiary structure predictions reported by Aloy and Russell [72], using the reported P-values as the internal measure of confidence in the interactions.

### Summary of integration

The final integrated gene network incorporates 10 fairly distinctive types of data: 1) small-scale protein physical interactions from literature curation, 2) co-citation evidence, 3) mRNA co-expression, 4) genetic interactions, 5) protein complexes derived from affinity-purification followed by mass spectrometry, 6) high-throughput yeast two hybrid analyses, 7) gene neighbors, 8) phylogenetic profiles, 9) Rosetta Stone protein linkages, and 10) inferred interactions from tertiary structural modeling (**Table 4**). The following pseudo-code summarizes the benchmarking and integration of these data:

1. For DNA microarray data
  1.1. For each set of yeast DNA microarrays (corresponding to all arrays from a given category defined in SMD)
    1.1.1. Calculate the mean-centered Pearson correlation coefficient (PCC) between all pairs of genes' expression profiles
      1.1.1.1. Calculate (by t-test) the minimum correlation coefficient for 99% confidence given the # of experiments in the set. For further analyses, consider only pairs meeting this criterion.
      1.1.1.2. Eliminate all potential cross-hybridization pairs defined by cDNA BLAST score (E-value<10^−4^ and nucleotide sequence identity >70%), then evaluate the regression between PCC and the log likelihood score (LLS) of sharing Gene Ontology biological process annotations
        1.1.1.2.1. Reject set if no relationship is evident between PCC and LLS
      1.1.1.3. Filter genes considered in the correlation analysis by requiring each gene to exhibit significant expression changes (e.g., >R-fold, typically ∼1.5-fold) in at least M microarray experiments across the data set. Optimize these 2 parameters by recall-precision analysis, maximizing the area under a plot of LLS versus # of genes participating in the linkages.
      1.1.1.4. Fit regression (typically sigmoid) between PCC and LLS, considering only genes passing the optimized filtering criteria (1.1.1.3) and only gene pairs whose correlation exceeds the 99% confidence level (1.1.1.1).
      1.1.1.5. Using regression fit, assign LLS scores to all gene pairs whose correlation exceeds the 99% confidence level, including unannotated gene pairs.
      1.1.1.6. Select minimum LLS threshold from inflection point of regression model. Retain only LLS scores/gene pairs surpassing threshold.
  1.2. Consider all sets of yeast DNA microarrays passing the filter of 1.1.1.2.1 as a single composite set and analyze as in 1.1 and subsections. (This step helps reconstruct linkages for globally co-expressed genes, such as housekeeping genes.)
  1.3. Integrate LLS scores from all analyses of DNA microarrays
    1.3.1. Calculate the weighted sum of LLS scores for each gene pair across the analyses of DNA microarray sets
    1.3.2. Optimize the choice of the weighting parameters D and T using recall-precision analysis by maximizing the area under a plot of LLS versus # of genes participating in the linkages. Compare to *naïve* Bayesian integration, and choose from weighted integration versus *naïve* Bayes by recall-precision analysis.
2. For each set of protein-protein physical interaction data (mass spectrometry analyses of purified complexes, genome-scale yeast 2 hybrid analysis, small-scale data collected by literature curation) and genetic interaction data (collected by literature curation)
    2.1. Fit regressions between LLS and data-intrinsic scores (–log(hypergeometric probability of interaction))
    2.2. Using regression fit(s), assign LLS scores to all interacting gene pairs, including unannotated gene pairs
    2.3. Integrate LLS scores from homogeneous types of detection methods using weighted sum method (e.g., integrate LLS from three major mass spectrometry analysis of complex [52]–[54] into a single integrated set of gene linkages from all mass spectrometry analyses), optimizing D and T parameters by recall-precision analysis. Compare to *naïve* Bayesian integration, and choose from weighted integration versus *naïve* Bayes by recall-precision analysis.
3. For co-citation, phylogenetic profiles, Rosetta-stone proteins, gene neighbors data, and inferred protein interactions from protein tertiary structure
    3.1 Fit regressions between LLS and data-intrinsic scores (–log(random probability of co-citation), mutual information of phylogenetic profiles, (–log(random probability of gene-fusion), –log(random probability of being gene neighbors, and original P score as in [72], respectively)
    3.2 Using regression fit(s), assign LLS scores to all co-cited (or co-inherited or co-neighboring) gene pairs, including unannotated gene pairs
4. Integrate all linkages using the weighted sum method, optimizing the choice of D and T parameters by recall-precision analysis. Compare to *naïve* Bayesian integration, and choose from weighted integration versus *naïve* Bayes by recall-precision analysis.

### Experimental validation of yeast ribosomal biogenesis genes

Yeast strains were cultured in YPD (1% yeast extract, 2% peptone, 2% dextrose) at either 20°C or 30°C. The *puf6Δ* haploid MATa deletion strain [36] and *PUF6*, *NMD3*, and *TDH1* TAP-tagged haploid MATa strains [40] were obtained from Open Biosystems.

For polysome profile analysis, yeast strains were cultured to OD_600_ 0.3–0.5, and 100 µg/ml cycloheximide (Sigma) was added to each culture. Cultures were immediately cooled with ice, and all subsequent steps were performed on ice or at 4°C. Each cell pellet was washed once with lysis buffer (20 mM Tris pH 7.4, 20 mM KCl, 5 mM MgCl_2_, 100 µg/ml cycloheximide, 12 mM β-mercaptoethanol). The cells were pelleted, resuspended in one volume lysis buffer with protease inhibitors (2 µg/ml leupeptin, 2 µg/ml aprotinin, 1 µg/ml bestatin, 1 µg/ml pepstatin A; obtained from MP Biomedicals Inc.), and lysed with glass beads. Crude lysates were centrifuged at 15,000g for 10 minutes. Fifteen OD_260_ units of each supernatant were loaded onto continuous 12 ml 7 to 47% sucrose gradients in lysis buffer without protease inhibitors, as in [73]. After a 2.5-h spin at 40,000 rpm in a Beckman SW40 rotor, the sucrose gradient was fractionated and absorbance at 254 nm was measured. For TAP-tagged strains, fractions were collected, and proteins were precipitated with 10% cold trichloroacetic acid and washed with 100% cold acetone.

For analysis of co-sedimentation with ribosomes, precipitated proteins were resuspended in 20 µl Laemmli buffer, and 2 µl of each sample was deposited onto a nitrocellulose membrane. TAP-tagged proteins were detected with a PAP antibody (Rockland Immunochemicals, Inc.) and electrochemiluminescence (ECL; GE Amersham).
